# Integrating an algorithmic and health systems thinking approach to improve the uptake of government antenatal nutrition services in Vidisha, Madhya Pradesh (India), 2018 to 2021

**DOI:** 10.1093/heapol/czad011

**Published:** 2023-02-06

**Authors:** Vani Sethi, Archana Mishra, K S Ahirwar, A P Singh, Sameer Pawar, Pushpa Awasthy, Ankita Tiwari, Avi Saini, Narendra Patel, Abhishek Kumar, Tashi Choedan, Mansi Shekhar, William Joe

**Affiliations:** Regional Nutrition Specialist, UNICEF Regional Office for South Asia, P.O.Box 5815, Leknath Marg, Kathmandu, Nepal; Maternal Health Division, Government of Madhya Pradesh, Arera Hills, Bhopal, Madhya Pradesh 462027, India; Chief Medical Health Officer, Government of Madhya Pradesh, Vidisha District, Bhopal, Madhya Pradesh 464001, India; Chief Medical Health Officer, Government of Madhya Pradesh, Vidisha District, Bhopal, Madhya Pradesh 464001, India; Public Health and Nutrition, UNICEF Field Office for Madhya Pradesh, Krishna Nagar, Shymala Hills, Bhopal, Madhya Pradesh 462002, India; UNICEF Field Office for Madhya Pradesh, Krishna Nagar, Shymala Hills, Bhopal, Madhya Pradesh 462002, India; UNICEF Field Office for Madhya Pradesh, Krishna Nagar, Shymala Hills, Bhopal, Madhya Pradesh 462002, India; Institute of Economic Growth, Delhi University Enclave (North Campus), New Delhi 110007, India; Institute of Economic Growth, Delhi University Enclave (North Campus), New Delhi 110007, India; Institute of Economic Growth, Delhi University Enclave (North Campus), New Delhi 110007, India; Institute of Economic Growth, Delhi University Enclave (North Campus), New Delhi 110007, India; Masters in Nutrition, PGDHM, Nutrition International Field Office for Madhya Pradesh, Basant Kunj, Shahpura, Bhopal, Madhya Pradesh 462016, India; Institute of Economic Growth, Delhi University Enclave (North Campus), New Delhi 110007, India

**Keywords:** Maternal nutrition, health systems, India, Vidisha, antenatal care

## Abstract

In 2018, the Government of Madhya Pradesh initiated the feasibility testing of integrating an algorithmic approach (assess, give, counsel, treat) to strengthen antenatal nutrition services in routine government-funded programmes coupled with a health system thinking approach to strengthen the health service delivery platform. Implementation phases included (1) an evidence review and stakeholder consultations (April 2018) and (2) a health systems strengthening preparedness phase (May–December 2018), including pilot testing in Vidisha district (January–December 2019) covering ∼54 100 pregnant women with 237 antenatal contact points through 241 government auxiliary nurse midwives/staff nurses. During 2020–21, feasibility testing was expanded to an additional 7 districts. We used programme registers of the Auxiliary Nurse Midwives Registers (2019–21) and National Family Health Survey data for 2016 and 2021 to show changes in the Vidisha district and 7 expansion districts. We compare the performance of Vidisha district with Ashok Nagar district, where no such intervention occurred. Comparing 2016 and 2021 data, the Vidisha district showed improvements in receipt of antenatal care in the first trimester (29 to 85%) and in four antenatal visits (17 to 54%). Using the difference-in-difference approach, a 42% net increase in first-trimester antenatal check-ups in Vidisha as compared to Ashok Nagar is observed. There was also an improvement in the maternal nutrition budget of the state from USD 8.5 million to USD 17.8 million during this period. The Vidisha initiative offers several lessons in time-effective workflow to deliver all constituents of nutrition services at various antenatal contact points through and via routine government health systems. Continued execution of the algorithm for screening, with longitudinal data on the management of all nutrition risks, will be critical to show its long-term impact on maternal morbidities and birth outcomes.

Key messages

- The study offers an important contribution to the implementation science around the impact of integrating an algorithmic (assess, give, counsel, treat) and a health systems thinking approach.
- We observed improvements in receipt of antenatal care in the first trimester and in four antenatal visits and reduction in anaemia in women of reproductive age in the intervention area.
- The algorithm-based protocol seems an effective strategy that simplifies the workload of the auxiliary nurse midwives and ensures targeted delivery of services and counselling.

## Introduction

The Indian government offers a basket of antenatal nutrition services to 30 million pregnant women at any given point in time (Government of India, 2018). These services include (1) nutrition assessment (height, weight, haemoglobin, gestational weight-gain monitoring), (2) provision of micronutrient supplements [iron and folic acid (IFA): 60 mg of iron and 500 µg of folic acid (Ministry of Health and Family Welfare, MoHFW, 2018], calcium supplements with 500 mg of elemental calcium and 250 international units of vitamin D (Ministry of Health and Family Welfare, MoHFW, 2014) and peri-conception folic acid (Ministry of Health and Family Welfare, MoHFW, 2018), (3) deworming tablets (400 mg of albendazole) (Ministry of Health and Family Welfare, MoHFW, 2014), (4) nutrition counselling (including diet, breastfeeding, family planning), (5) unconditional cash transfers (85 USD per pregnancy) (Government of India, 2006), (6) insecticide-treated bed nets in malaria-prone areas, (7) screening and nutrition and/or medical management of fluorosis, anaemia, tuberculosis, gestational diabetes mellitus and malaria, and (8) balanced energy protein supplementation in the form of a take-home ration/hot cooked meal, comprising 600 calories and 18–20 g of protein, 25 days per person per month. These services are delivered through centrally sponsored flagships of two Ministries—Integrated Child Development Services (Ministry of Women and Child Development) and National Health Mission (Ministry of Health and Family Welfare). In addition, state governments have initiated and funded schemes for improving maternal nutrition services through innovative approaches—maternal spot-feeding programmes (Sethi *et al*., 2019b; Kachwaha *et al.*, 2021) and engagement with women self-help groups for screening and support of nutrition risks (Shrivastav *et al.*, 2021) and conditional cash transfer schemes. For example, in the State of Madhya Pradesh, an additional USD 213 is provided to pregnant women for delivery at health facilities (Government of India, 2006).

Despite programmes, coverage of antenatal nutrition services in India remains constrained by several programmatic challenges (Kulkarni *et al.*, 2016). To name but a few, (1) priority is still accorded to reducing maternal mortality and not morbidity, hence the focus remains on maternal severe anaemia and not maternal thinness and maternal obesity (Say and Chou, 2018), (2) there is a lack of operational know-how on time-effective workflow to deliver all constituents of nutrition services at various antenatal contact points (facility, outpatient antenatal clinics, outreach village outposts) (Jiwani *et al.*, 2020), and (3) standard and simplified job aids tailored to gestational month as well as the type of nutrition risk are missing. This creates constraints for the health workers on what to ask, how to classify nutritional risk and what to do when a risk is identified. (4) When nurses’ and health providers’ training takes place, the nutrition component is weak/missing (DiMaria‐Ghalili *et al.*, 2013). (5) A cadre of trainers who understand nutrition and dietetics to support the nutrition component pieces of training in medical training is also lacking. (6) Women who are thin, short, anaemic, obese and with depression require ‘extra care’ (Higgins *et al.*, 2018; Lawrence *et al.*, 2020). This extra care is often absent not because of intent but due to lack of clarity for their systematic screening and management. (7) At the planning level, all nutrition items (supplies, training, human resources, cadre, monitoring and research) are not/inadequately budgeted for in the annual health budget plans (Tsofa *et al.*, 2021; Saini *et al.*, 2022). (8) At reporting and review level, a consensus on which critical nutrition indicators to include is either unavailable or often lacking, even in recording registers and reporting, and as a result this is never reviewed (Adhikari and Bredenkamp, 2009). (9) Maternal nutrition rapid assessment questions are missed when antenatal care (ANC) rapid assessments are carried out.

In Madhya Pradesh, as per the National Family Health Survey (NFHS) 2019–21 data, only 58% of women received four or more ANC visits while only 50% reported consuming IFA for ≥100 days during their last pregnancy. These figures are relatively poor, with the coverage of these indicators being 70–90% across most of the well-performing states (International Institute of Population Sciences, 2021). In view of this, the Government of India and the Government of Madhya Pradesh developed and tested algorithms, with operational guidelines, which support health workers across routine antenatal contact points to conduct nutrition assessments and gestational month-specific nutrition counselling, give nutrition micronutrient supplements, classify nutrition risks as well as provide extra counselling for those with nutrition risks, and on other hand strengthen all aspects of health systems thinking to deliver nutrition services (supplies, information systems, financing, communication, governance).

In this paper, we describe the processes adopted. We use government district health management information systems programme data and NFHS data for 2015–16 (as the baseline) and 2019–21 (as the endline) to compare changes in the Vidisha district. We also use the Vidisha district (intervention district) and compare this with Ashok Nagar (comparison district with similar typography and no such intervention) to draw insights regarding the effect of this intervention.

## Methodology

### Arriving at an algorithm

The release of the World Health Organization (WHO) 2016 guidelines for positive pregnancy experience (World Health Organization, 2016) and results of the fourth round of NFHS, 2016 (NFHS-4) served as a trigger for the Government of India to host a series of expert consultations in 2017 to review the evidence and programme options to strengthen the nutrition services in ANC as well as achieve clarity on the 14 nutrition interventions of the WHO 2016 guidelines. A sub-committee on maternal nutrition, which included diverse professionals from dietetics, public nutrition, gynaecology and paediatrics, development partners, academics and state representatives (Madhya Pradesh) was formed. This group developed algorithms and job aids and facilitated testing them in various hospitals with funding support from UNICEF (Sethi *et al*., 2019a; Choedon *et al.*, 2021). Further testing was carried out at various sites like nutrition rehabilitation centres, infant and young child feeding centres, outpatient departments antenatal clinics and Pradhan Mantri Surakshit Matratav Abhiyaan (the 9th day of every month dedicated to ANC), using an additional cadre of nutritionists from a medical college to deliver the package with the health worker. Once these materials were ready, it was necessary that they be used in real-time routine government systems and with government budgets at health facilities and at village health and sanitation level, by health functionaries including auxiliary nurse midwives (ANMs) without any additional support from professional nutritionists.

The developed algorithms were piloted in Madhya Pradesh and then fine-tuned to state context. The final versions can be seen in Figures S1 and S2, see online supplementary material. It was the workflow in the algorithms that was tested for programme feasibility of use at scale as part of a routine ANC programme. As the ANM/staff nurse registers the pregnant women and does routine ANC assessments, she now had to also compulsorily conduct and record nutrition assessment [height, mid-upper arm circumference (MUAC) and body mass index (BMI], give micronutrient supplements (IFA and calcium tablets), deworm and provide gestational monthly counselling. Then, risk is then classified risk based on nutrition assessment. Those pregnant women who are at nutritional risk (short, thin, young, anaemic, obese) are included as high-risk pregnancies. The ANM would provide nutrition risk-specific counselling using the counseling cards. As a follow-up, community-level workers such as accredited social health activists (ASHA) and or Anganwadi workers (AWW) will conduct a monthly follow-up visit and provide individual nutritional risk-based counselling and motivation for compliance in the consumption of micronutrients. If there are severe nutrition risks, the pregnant woman is referred to a medical officer in the nearest health facility.

### Applying health systems to strengthen the platforms

#### Leadership and team preparedness phase (April–December 2018)

Between May–December 2018 an organization of the Government of Madhya Pradesh provided the anchor leadership and brought in local partners to develop state operational guidelines and a monitoring framework and conduct a gap assessment of health systems to deliver nutrition interventions ably. This included the availability of supplies (digital haemoglobin metres, adult MUAC tapes, gestational-month-specific and nutrition-risk-specific counselling aids and glucometers), gaps in recording and reporting of nutrition interventions, training needs of health workers, and additional financing for training and materials. On the basis of the assessment, a rollout plan was developed which included state operational guidelines, local translation of existing training package (algorithm, counselling aids), prepositioning of supplies, preparation for capacity building (training package, training plan), development of reporting and a rapid assessment checklist and indicators for review.

#### Pilot phase and expansion phase

**Figure 1.**
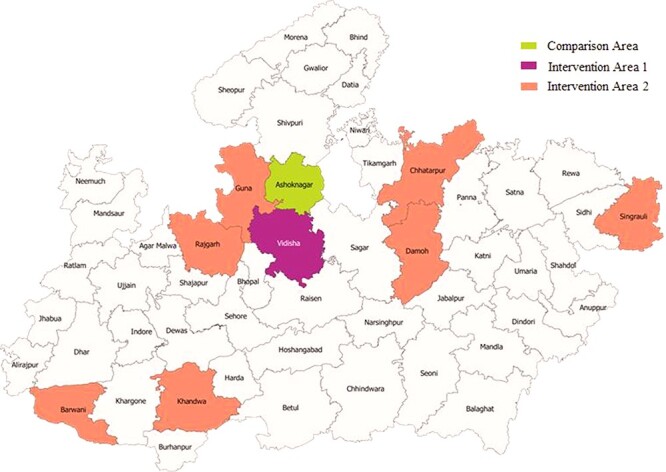
Map of the study area, Madhya Pradesh

Note: Intervention area 1 (pilot district, Vidisha district), intervention area 2 (data for expansion districts, 7 districts) and comparison area (Ashok Nagar, not a pilot and not an expansion district).

Madhya Pradesh has 52 districts (covering an estimated 2.2 million pregnant women) (Figure 1). Of the 52 districts, 8 are socio-economically backward with poorer health indicators and hence receive additional focus and are identified as ‘aspirational districts’ under a national programme for backward districts (NITI Aayog, 2018). These include East Nimar, Rajgarh, Barwani, Chhatarpur, Vidisha, Singrauli, Damoh and Guna. The pilot district that would test the programme feasibility of the twin intervention (algorithms and health systems strengthening) was agreed to be Vidisha district (an aspirational district at 57 km distance from the state headquarters). The scope of the pilot phase in Vidisha district was to adopt the algorithmic approach when providing services to an estimated 54 100 pregnant women through 31 antenatal clinics in facilities and 206 village health and nutrition day outreach centres by 206 ANMs and 35 staff nurses. The on-ground pilot testing in Vidisha district was between January–December 2019. Here, training and monitoring were done with additional support from UNICEF as these items were not included as part of state government budgets. From January 2020 till December 2021 the algorithmic approach with costs was included as part of government budgets and the programme feasibility testing was expanded to 7 additional aspirational districts. In 2022, the initiative continues in a total of 8 districts (pilot and expansion phase) and involves 2163 health workers (Table S2, see online supplementary material).

#### Capacity building of health providers

In 2019, in the pilot phase in Vidisha district, national master trainers (2 people) trained state master trainers (6 people) who in turn conducted cascade training for 226 health personnel including medical officers, ANMs and staff nurses of Vidisha district in district hospitals, block hospital/centres and primary health centres. Each training session was of 15 h duration (over 3 days). The training covered the algorithm as well as supporting gap assessment and arriving at corrective actions for strengthening health systems The national master trainers included those who had implemented the package in a medical college in Delhi. After the first round of training, the next round was organized after 6 months. In the expansion phase (2020–21) training was conducted in online mode in view of the COVID pandemic. In the expansion phase, the master trainers included those who had implemented the intervention in the pilot district train the district-level trainers who in turn conduct training for the districts.

#### Generating demand

Several steps were taken as part of routine communication efforts to spread awareness for improving the uptake of antenatal services, e.g. radio/television communication through village-level meetings, etc. To ensure pregnant women comply with the advice, those mothers who were at nutrition risk were provided monthly counselling by health workers (ANM/ASHA) either in their homes or on the doorstep during the COVID-19 pandemic.

#### Additional financing

In 2019, the first year of the rollout, additional cost (over and above the cost of supplements, infrastructure, human resources, review, etc.) for this intervention was USD 29 518 which was funded by UNICEF. From 2020 onwards, the budget was included as part of the government budget and received sanction under maternal health and research and innovation (USD 88 671). There was also an improvement in the overall maternal nutrition budget of the state from USD 8.5 million to USD 17.8 million during this period (Table S1, see online supplementary material, and Table 1).

**Table 1.** Budget allocated under the National Health Mission, for roll out of the maternal nutrition programme in 7 aspirational districts of Madhya Pradesh, for years 2020–21

SN	Components	Budget (USD)
1	Procurement for scaling-up of maternal nutrition intervention in 7 aspirational districts	12 081
2	Review meeting for scaling-up of maternal nutrition intervention in 7 aspirational districts	1342
3	One-day state-level sensitization cum orientation workshop for scaling-up of maternal nutrition intervention in 7 aspirational districts	671
4	One-day training at district-level for functionaries from 356 facility (Medical Officer (MO)/Staff Nurse (SN)/Auxiliary Nurse Midwife (ANM)/counsellors) of 7 aspirational districts for scaling-up of maternal nutrition intervention	6349
5	Two days training of 1585 outreach ANMs for scaling-up of maternal nutrition intervention in 7 aspirational districts	38 685
6	One-day district-level orientation of health supervisors, MPW, ASHA Sahyogini and ICDS supervisors for scaling-up of maternal nutrition intervention in 7 aspirational districts	20 537
7	All maternal health registers (Information Education Communication (IEC) Material)	9007
Total		88 672

Source: UNICEF Madhya Pradesh based on Record of Proceedings, 2020–21, Ministry of Health and Family Welfare. Exchange rate as per 9 February 2022 taken as 1 INR = 0.0134 USD, source: India foreign exchange rate Reserve Bank of India.

#### Reporting, monitoring and review

The routine health systems reporting mechanism is used. The ANM updates the details about the health and nutrition services received by the pregnant mothers in the Reproductive and Child Health (RCH) register, available with health care worker and Mother and Child Protection (MCP) card, given to pregnant women and mothers. Manual reports in the prescribed format are handed over to the health supervisor along with a Health Management Information System (HMIS, portal operated by Ministry of Health)-compiled report for those data fields that are not covered in the routine reporting system. Thereafter, the supervisor submits the compiled reports to the block coordinators. After submission of sub-centre-level manual reports, the existing data entry operator updates the reports at the block level. The compiled manual report from the block is then submitted to the district by the block programme manager. From the district, the report is compiled and submitted to the state by a monitoring and evaluation officer. Three (weight < 35 MUAC, BMI, nutrition risk classification) indicators are added to the RCH register. UNICEF supported external monitors [two for the district hired through the National Health Mission (NHM)] who were entrusted the responsibility of providing handholding support to the district health team. Biannual reviews of progress on the ground were held under state leadership, whereupon, based on field assessment on rollout, corrective actions were taken. Every Saturday, a meeting of all ANMs was held by each existing health supervisor where the ANMs were supported in updating the backlog data from their registers into online systems.

#### Supplies

Most of the supplies (like nutritional supplements, equipment) were available through government resources, while some of the newly introduced components (like MUAC tapes, training and counselling material) were arranged in collaboration with development partners. Digital haemoglobin metres (one per ANM) were provided by the Tata Trust. As part of capacity building, partner teams, including external monitors hired by UNICEF, provided handholding support to the store managers for better forecasting and inventory management.

#### Assessing change

We compared health systems performance and uptake of nutrition services across three areas—intervention area 1 (pilot district, Vidisha district), intervention area 2 (data for expansion districts, 7 districts) and comparison area (Ashok Nagar, not a pilot and not an expansion district) (Table S3, see online supplementary data). We used unit level data of the fourth and fifth rounds of the NFHS for 2015–16 (serving as the baseline) and 2019–21 (serving as the endline). We used three indicators: (1) numbers and timing of the first antenatal check-up to assess health systems strengthening, (2) consumption of IFN for ≥180 days to assess the update in nutrition services and (3) women with anaemia to assess change.

For these three areas, we also used government district HMIS data for the yearly periods September 2018, 2020 and 2021 to compare three indicators: (1) programme monitoring reporting efficiency, (2) stockouts of micronutrient supplement IFA and (3) receipt of micronutrients (IFA and calcium). We also used the RCH data from the population register for 2020 and 2021 which included information on women with weight <35 kg, severe anaemia and those receiving various micronutrient supplements such as folic acid and calcium. We also used the data for Vidisha district (intervention district) and compared this with Ashok Nagar. The district Ashok Nagar was selected as a comparison because no intervention took place in this area. Not only is Ashok Nagar adjacent to Vidisha but both districts are quite similar with respect to typography (Figure 1) and socio-economic characteristics. The district was selected after deliberation with personnel from the field office of UNICEF.

#### Statistical analyses

We used the difference-in-difference approach to show the net change in Vidisha district as compared to Ashok Nagar district. The differential effects were tested by using regression models that estimate changes over time between the two areas: intervention and comparison area. The rationale behind the difference-in-difference approach is that the two groups are expected to trend consistently over time. Here the counterfactual is that the implementation of the programme in the intervention area should be associated with an improvement in utilization of ANC services. The effects can be estimated using the following model where *Y* denotes the outcome-related variables (mothers who had an antenatal check-up in the first trimester/mothers who had at least four ANC visits/consumption of IFA for ≥180 days in pregnancy). *D*_1_ is the indicator variable for time period and assumes a value of 1 for the NFHS-5 (2021) period and 0 for NFHS-4 (2016). *D*_2_ is an indicator variable for the intervention area (Vidisha district) and is assigned a value of 1 for intervention area 1 (Vidisha) and 0 for comparison area (Ashok Nagar).


$${{\rm{Y}}_{\rm{t}}} = {\beta}_1^{\rm{\,}} + {\beta}_2^{\rm{\,}}{{\rm{D}}_1} + {\beta}_3^{\rm{\,}}{D_2} + {\beta}_2^{\rm{\,}}{{\rm{D}}_1}{{\rm{D}}_2} + {\mu}_{\rm{t}}^{\rm{\,}}$$

To elaborate, the product of *D*_1_ and *D*_2_ refers to the impact of the programme in an intervention area over time. In addition, based on a literature review we identified seven key correlates of utilization of antenatal services, i.e. place of residence, education and age of women, wealth, number of children, social group and religion (Singh *et al.*, 2012; Saad–Haddad *et al.*, 2016; Okedo-Alex *et al.*, 2019). Apart from unadjusted estimates, difference-in-difference estimates were calculated after adjusting for all these socio-economic correlates using the ‘diff’ command in Stata 15. The distribution of demographic and socio-economic characteristics is presented in supplementary Table S4, see online supplementary material.

Similarly, difference-in-difference estimates for variables related to RCH data from the population register, which included information on women with weight <35 kg, severe anaemia and women receiving various micronutrient supplements such as folic acid and calcium obtained from the RCH register, were calculated manually as only aggregate-level data were available from these databases.

In addition, the annual average rate of reduction (AARR), which is the average relative percent decrease per year in prevalence or rate (World Health Organization, 2021), was calculated as follows. Here ‘*n*’ is the number of years between the time periods denoted by ‘*t*’ and ‘*n* + *t*’ and *P*_t+n_ and *P*_t_ are the prevalence rates at time points ‘*t* + *n*’ and ‘*t*’ respectively. The data from the NFHS were available for 2015–16 and 2019–21, while data from the HMIS were available for 2018–21 and for the RCH register for 2020 and 2021.


$$AAR{R_{\rm{\,}}} = (1 - ( {{\raise0.7ex\hbox{${{\rm{\,P}}_{{\rm{t}} + {\rm{n}}}^{\rm{\,}}}$} \!\mathord{\left/{\vphantom {{{\rm{\,P}}_{{\rm{t}} + {\rm{n}}}^{\rm{\,}}} {{\rm{P}}_{\rm{t}}^{\rm{\,}}}}}\right.-}
\!\lower0.7ex\hbox{${{\rm{P}}_{\rm{t}}^{\rm{\,}}}$}}{)^{1/n}}} )$$

A positive sign of AARR indicates a reduction or downward trend, while a negative sign indicates an increase, or upward trend.

The annual average rate of increase (AARI) which is the average relative percent increase per year in prevalence or rate was calculated as


$$AAR{R_{\rm{\,}}} = (( {{\raise0.7ex\hbox{${{\rm{\,P}}_{{\rm{t}} + {\rm{n}}}^{\rm{\,}}}$} \!\mathord{\left/{\vphantom {{{\rm{\,P}}_{{\rm{t}} + {\rm{n}}}^{\rm{\,}}} {{\rm{P}}_{\rm{t}}^{\rm{\,}}}}}\right.-}
\!\lower0.7ex\hbox{${{\rm{P}}_{\rm{t}}^{\rm{\,}}}$}}{)^{\frac{1}{n}}} - 1} )$$

A positive sign of AARI indicates an increase or upward trend, while a negative sign indicates a decrease or downward trend.

## Results

### Service uptake in Vidisha district

Comparison of the fourth (2016) and fifth (2021) rounds of NFHS data for Vidisha shows improvement in footfall for receipt of ANC in the first trimester (29 vs 85%) and receipt of four antenatal visits (17 vs 54%). Data also show that compliance in consumption of IFA tablets for ≥180 days in pregnancy (4 vs 25%) has significantly improved. Overall anaemia was lowest in Vidisha district compared to other districts in Madhya Pradesh in 2021, and between 2016 and 2021 the proportion of anaemic women in Vidisha was reduced from 44.2 to 38.5% (Table 2 and S5, see online supplementary material).

**Table 2.** Status of maternal services in pilot district (intervention area 1), expansion districts (intervention area 2) and non-expansion district (control) of Madhya Pradesh

	Mothers who had an antenatal check-up in the first trimester (%)			Mothers who had at least four ANC visits (%)			Consumption of ≥IFAfor 180 days in pregnancy (%)		
**District**	**NFHS-4,** **2016**	**NFHS-5,** **2021**	**AARR** **/AARI**	**NFHS-4,** **2016**	**NFHS-5,** **2021**	**AARR** **/AARI**	**NFHS-4,** **2016**	**NFHS-5,** **2021**	**AARR** **/AARI**
Vidisha (area 1)	29.5	84.9	23.5	16.9	54.4	26.3	4	24.9	44.2
	[22.7,37.4]	[79.4,89.1]		[11.4,24.3]	[43.4,65.1]		[2.0,7.8]	[18.2,33.1]	
Rajgarh (area 2)	52.2	75.1	7.5	35.9	55.1	8.9	5.4	28.5	39.5
	[45.1,59.2]	[67.7,81.2]		[27.1,45.9]	[46.1,63.8]		[3.2,9.0]	[22.2,35.7]	
Barwani (area 2)	42.7	88.8	15.8	26.3	64.6	19.7	12.5	33	21.4
	[34.2,51.6]	[83.3,92.7]		[19.4,34.6]	[57.4,71.1]		[8.5,17.9]	[25.1,42.0]	
Khandwa (area 2)	73	65.5	2.1	48.5	62.2	5.1	9.2	27.6	24.6
	[66.9,78.3]	[52.3,76.7]		[41.5,55.7]	[48.5,74.2]		[6.2,13.5]	[16.2,42.8]	
Damoh (area 2)	31.1	67.1	16.6	24.2	46.4	13.9	7.3	29.1	31.9
	[24.0,39.1]	[59.0,74.2]		[19.1,30.2]	[38.3,54.7]		[4.4,12.0]	[22.9,36.1]	
Chhatarpur (area 2)	36.2	65.8	12.7	19.4	36.9	13.7	3.8	15.6	32.6
	[31.0,41.8]	[59.0,72.0]		[14.1,26.1]	[30.4,43.8]		[2.3,6.4]	[9.7,24.1]	
Singrauli (area 2)	29.2	72.9	20.1	20.9	58.1	22.7	1.7	21.7	66.4
	[22.8,36.5]	[63.2,80.9]		[15.8,27.3]	[48.1,67.5]		[0.6,4.5]	[14.8,30.8]	
Guna (area 2)	60.6	84.6	6.9	31.9	68.7	16.6	3.8	43.4	62.8
	[52.2,68.5]	[77.8,89.7]		[24.6,40.3]	[62.2,74.5]		[1.8,8.1]	[36.2,51.0]	
Ashok Nagar district (control)	68.3 [60.1,75.5]	81.8 [73.9,87.7]	3.7	38.5 [29.0,49.0]	58.3 [47.7,68.3]	8.7	7.2 [4.2,11.9]	26.6 [19.9,34.5]	29.9
Unadjusted difference-in-difference		41.85*** [31.8,51.9]			15.22*** [4.2,26.2]			0.4 [−7.5,8.3]	
Adjusted difference-in-difference		39.56*** [29.6,49.5]			11.63** [0.7,22.5]			−0.91 [−8.9,7.1]	

Note: difference-in-difference calculated as net improvement in intervention area over control area between 2016–17 and 2021–22. Figures in parenthesis are 95% confidence interval. Level of significance- ** *P* < 0.01, *** *P* < 0.001.

### Changes in health systems to deliver maternal nutrition services

Several items missing in 2018 were integrated as part of the government antenatal services both from an intervention as well as systems perspective (Table 3). The missing indicators were integrated into the existing record-keeping and reporting mechanism. Budgeting-wise, there were considerable improvements as several missing items which previously were not budgeted for were now included in the budgets by government systems (digital haemoglobin metres, MUAC tapes, nutrition counselling materials). There was also an increase in the overall maternal nutrition budget of the state from USD 8.5 million to USD 17.8 million during this period. After 1 year, interviews with ANMs (*n* = 52) from Vidisha district showed that 95% of the ANMs had implemented the algorithm; 77% reported it had not increased their workload but aided streamlining of the work flow. Yet, only 45% of ANMs could provide the correct description of the all steps of the algorithm, with 55% reporting measuring and recording of MUAC of the pregnant women, showing that repeat training and handholding is imperative.

**Table 3.** Applying health systems thinking to assess preparedness of Vidisha District to deliver maternal nutrition services

Health systems pillar	2018	2021
**Leadership and governance** Operational guidelines in place for strengthening maternal nutrition Nutrition risk assessment (thinness and overweight), classification and counselling included as part of antenatal care MUAC and BMI as part of nutrition assessment Iron and folic acid supplements	**X** **X** **X** **100 mg**	**√** **√** **√** **60 mg**
**Capacity building** State-specific comprehensive ANC including maternal nutrition-training module and counselling material, gestational month-wise and nutrition-risk-specific cards, recipe book for maternal severe thinness and obesity Training of field staff	**X** **X**	**√** **√^a^**
**Partnerships** State-level thematic technical working group/committee for maternal nutrition Joint planning, monitoring, review and feedback to government	*X* *X*	√ √
**Supplies** Availability of digital haemoglobin metres Availability of MUAC tapes Supply managers supported in planning and monitoring stockouts	*X* *X* *X*	**√** **√** **√**
**Financing (USD)** Maternal nutrition training Maternal nutrition counselling materials Overall maternal nutrition budget Nutrition services monitoring and handholding support	0 0 8.53 million 0	657 000 20 950 17.8 million 20 000
**Information systems—indicators missing that are now included** Pregnant Women ‘at nutrition risk/medical risk’ Pregnant Women with any nutritional risk counselled Pregnant Women having MUAC < 23 cm Pregnant Women having BMI (up to 20 weeks) < 18.5 kg/m^2^ Pregnant Women with night blindness/fluorosis/goitre	X X X X X	**√** **√** **√** **√** **√**

a Face-to face in 2019 and online in 2020–21.

### Changes in uptake of nutrition services based on programme data

Data from HMIS show that during the intervention there was no stockout of micronutrients (IFA tablets, albendazole and calcium tablets) in the district drug store between the periods 2018–19 and 2021–22. Vidisha district shows improvement in receipt of IFA (80 vs 90%) and calcium tablets (66 vs 69%) between the years 2018 and 2020. However, the proportion of women who received both micronutrient supplements (IFA and calcium tablets) was lower in Vidisha and Ashok Nagar district in 2021 as compared to 2020, which could possibly be due to the pandemic which forced the community workers to perform COVID-related services (Table 4). Also, while data are now being included in the RCH register, the reporting efficiency was sub-optimal and there was also a huge discrepancy between the RCH and the HMIS reported data. This shows that investment in reporting efficiency requires strengthening (Table 5).

**Table 4.** Reporting efficiency and receipt of maternal services in pilot district (intervention area 1), expansion districts (intervention area 2) and non-expansion district (control) of Madhya Pradesh—HMIS cumulative data for years 2018–21

	AARI/AARR					
	2018	2020	2021	2018 to 2020	2020 to 2021	2018 to 2021
Reporting efficiency						
Vidisha (area 1)	82.8	78.9	69.0	2.4	12.5	5.9
Rajgarh (area 2)	84.2	73.4	64.5	6.6	12.1	8.5
Barwani (area 2)	95.0	95.0	92.3	0.0	2.8	1.0
Khandwa (area 2)	82.9	95.0	95.0	7.0	0.0	4.6
Damoh (area 2)	90.4	85.6	63.4	2.7	25.9	11.2
Chhatarpur (area 2)	86.6	85.3	73.3	0.8	14.1	5.4
Singrauli (area 2)	95.0	95.0	95.0	0.0	0.0	0.0
Guna (area 2)	80.8	74.8	62.4	3.8	16.6	8.3
Ashok Nagar (control)	82.9	83.1	77.7	0.1	6.5	2.1
Receipt of IFA						
Vidisha (area 1)	79.9	90.4	78.4	6.4	13.3	0.6
Rajgarh (area 2)	76.5	70.9	66.1	3.7	6.8	4.8
Barwani (area 2)	78.3	84.6	78.1	3.9	7.7	0.1
Khandwa (area 2)	74.3	90.9	93.9	10.6	3.3	8.1
Damoh (area 2)	81.0	74.5	68.0	4.1	8.7	5.7
Chhatarpur (area 2)	79.0	85.2	73.2	3.8	14.1	2.5
Singrauli (area 2)	95.0	95.0	93.4	0.0	1.7	0.6
Guna (area 2)	76.7	68.8	59.2	5.3	14.0	8.3
Ashok Nagar (control)	76.9	78.0	75.8	0.7	2.8	0.5
Receipt of calcium tablets						
Vidisha (area 1)	66.0	69.4	65.3	2.5	5.9	0.4
Rajgarh (area 2)	57.1	70.3	72.6	11.0	3.3	8.3
Barwani (area 2)	73.0	89.9	93.1	11.0	3.6	8.4
Khandwa (area 2)	79.5	74.0	64.1	3.5	13.4	6.9
Damoh (area 2)	78.1	84.9	73.3	4.3	13.7	2.1
Chhatarpur (area 2)	80.5	88.3	87.9	4.7	0.5	3.0
Singrauli (area 2)	74.9	64.3	58.7	7.3	8.7	7.8
Guna (area 2)	67.1	69.8	73.0	2.0	4.6	2.8
Ashok Nagar (control)	78.6	90.3	71.8	7.2	20.5	3.0

**Table 5.** RCH register programme data

District Name	IFA 180 tablets	Folic acid 30 tablets	Albendazole 1 tablet	Calcium 180 tablets	Severe anaemia	Weight < 35	BMI < 18	BMI > 23	Age < 19	3/5 Doses of iron sucrose
2020										
East Nimar (Khandwa)	50.0	11.1	31.1	56.3	1.1	18.5	0.1	0.0	2.5	1.8
Chhatarpur	6.8	3.0	35.9	13.6	2.4	8.8	0.1	0.0	3.8	0.8
Guna	5.2	2.7	27.0	26.9	0.9	7.5	0.0	0.2	0.8	0.4
Rajgarh	14.0	4.7	35.5	57.0	1.2	12.4	0.2	0.2	2.9	0.7
Barwani	18.3	5.5	38.2	24.5	3.1	12.7	0.0	0.0	9.1	5.2
Singrauli	11.9	7.5	33.3	12.3	6.3	7.1	0.0	0.0	3.1	0.2
Ashoknagar	12.2	4.0	34.9	37.9	1.8	11.3	0.0	0.0	1.8	0.8
Damoh	12.1	3.3	35.0	33.4	1.0	13.3	0.0	0.9	5.0	1.5
Vidisha	13.2	3.3	32.2	55.0	0.6	8.9	0.1	0.5	2.6	1.0
2021										
East Nimar (Khandwa)	66.3	13.5	84.8	100	0.5	26.3	1.0	4.0	3.4	14.8
Chhatarpur	9.4	4.2	100	54.8	1.8	13.6	2.4	2.1	3.2	16.5
Guna	15.6	8.5	87.3	100	0.7	12.4	1.0	4.0	1.2	9.5
Rajgarh	29.9	10.4	81.3	100	0.8	18.1	0.9	1.4	2.5	12.4
Barwani	28.3	9.2	84.4	93.2	1.7	30.1	0.4	0.4	7.6	57.1
Singrauli	16.2	8.7	100	42.0	1.5	8.7	0.2	1.4	2.5	5.5
Ashoknagar	16.4	4.6	99.1	100	1.7	24.0	0.4	3.4	1.8	8.8
Damoh	27.0	9.2	90.2	100	1.0	17.5	1.0	4.9	4.6	8.9
Vidisha	17.5	8.7	67.4	100	0.7	15.1	0.7	4.2	1.8	9.0
Difference-in-difference over base year										
Vidisha	4.3	5.4	35.2	103.3	0.0	6.2	0.6	3.7	−0.8	8.0
Ashok Nagar	4.3	0.7	64.2	74.5	−0.1	12.7	0.4	3.4	0.0	8.0
Net change	−0.01	4.70	−28.97	28.82	0.13	−6.47	0.22	0.36	−0.80	0.06

There were challenges as well. ANMs transferred from the facility took away the counselling material along with them, a few ANMs misplaced the material, there were high caseloads at facilities, limited human resources, frequent turnover of staff, gaps in the routine reporting system, lack of motivation leading to non-adherence to the directives, and weak district and block review systems. Frequent changes in duties necessitated that all the staff nurses be trained. In addition to HMIS data, the uptake of nutrition services (such as IFA, folic acid, albendazole and calcium tablets) was calculated using RCH register data.

### Changes in expansion districts

The government initiated expansion in 2020 to an additional 7 districts using government funds, with the intention of covering an estimated 338 700 pregnant women through 237 antenatal health facilities and 1585 sub-health centres. At present only one round of training has taken place. NFHS data show a >30% increase in receipt of ANC in the first trimester across Barwani, Damoh and Singrauli. Also, a >10% reduction in women who are thin is reported across Barwani, Singrauli and Guna. Evaluation of the potential impact of the intervention in the expansion phase is warranted before scaling-up across the remaining districts. However, overall changes in the expansion districts are not as apparent as in Vidisha.

### Comparing Vidisha and Ashok Nagar districts

The difference-in-difference approach was used to assess the net change in Vidisha district as compared to Ashok Nagar district. More than 40% net increment [95% confidence interval (CI):31.8,51.9] in mothers who registered for an antenatal check-up in the first trimester and a 15% [95% CI: 4.2,26.2] increase in four ANC visits was observed in Vidisha district. Also, a significant decline in the percentage of women who are thin is observed across both districts. Notably, the prevalence of anaemia decreased from 44.5 to 38.2% in Vidisha but increased in Ashok Nagar from 42.3 to 46.1%.

## Discussion

The relevance of the comprehensive ANC package implemented in Vidisha district, to identify all types of nutrition risks, classify them and provide contextualized counselling and services, is underscored by the fact that women in Vidisha district continue to enter pregnancy with various nutrition risks including thinness (23%), obesity (20%) and anaemia (38%) (International Institute of Population Sciences, 2019–21) (Table S5). Hence nutrition-risk-specific counselling along with general nutrition and diet counselling is critical. This study offers an important contribution to the implementation science around strengthening nutrition services integrated in ANC.

Our major findings on the impact of integrating an algorithmic (assess, give, counsel, treat) and health systems thinking approach are as follows. First, our analysis reveals that nutrition service utilization such as receipt of ANC in the first trimester, receipt of four antenatal visits and consumption of IFA tablets for 180 days during pregnancy in Vidisha district has shown a significant improvement. Second, data also shows an increase in receipt of ANC in first trimester and improvements in anaemia levels across Barwani, Damoh and Singrauli. This is probably attributable to the trained health workers who were able to counsel the pregnant women and strengthened the reporting mechanism by updating the MCP cards. In addition, Barwani Damoh and Singrauli are aspirational districts. Sometimes review and monitoring from leader and district collectors can lead to improvements in service delivery. Deeper qualitative assessment will be required to gain an in-depth understanding of this progress. In addition, the improvement in anaemia seems to be consistent with the improvement in uptake of IFA tablets as observed in HMIS data. Recently, a study reported that training and sensitization of state and district officials could be instrumental in increasing the uptake of micronutrient supplementation such as IFA during pregnancy, which is associated with a reduction in the prevalence of anaemia (Joe *et al.*, 2022). Notably, the progress across East Nimar with respect to those who had a first antenatal check-up in the first trimester has been subdued. Third, estimates from HMIS and population register data show the negative the impact of COVID, resulting in disruption of services as well as reporting deficiency. These findings also obtain support from some of the other small-area studies which report that COVID-19 disrupted the provision and use of health and nutrition services in Uttar Pradesh (Nguyen *et al.*, 2021). In fact, the analyses of district-level data also reveal that delivery of maternal and child health services was disrupted to a great extent though it was quickly restored after the lockdown period from March to June 2020 (Avula *et al.*, 2022).

Our findings are in line with other similar studies (Rajpal *et al.*, 2021) that confirm a positive impact of system strengthening activities on utilization of health and nutrition services. In this regard it is worth mentioning that although most births take place at government health facilities, it will take additional efforts to change dietary practices that are determined by a range of factors including food-related norms and culture. Nevertheless, there is ample scope to integrate this package into routine ANC platforms in other contexts as 77% of ANMs reported that the comprehensive package has helped them to serve better without necessarily increasing their workload.

It is worth mentioning that that the pandemic affected the delivery of maternal and child health services and could easily erode some of the gains of this intervention. For instance, even within Vidisha the performance is not uniform. HMIS data indicate that not all blocks in Vidisha benefitted equally, which calls for strengthening of programme expansion. This also calls for increased efforts into identifying the possible causes of lacklustre performance in the blocks where uptake of services has been low.

There are a few caveats to this study. The study focused only on pregnant women covered by the government ANC programmes receiving ANM interventions. However, pregnant women who avail of services from private sector or other traditional health providers were not covered. Also, the aim of the study was to establish the association between service utilization and changes in outcomes but not necessarily in a causal framework. The use of cross-section survey data and lack of a clinical trial set-up for the study disallows the attribution of direct causality. Third, HMIS data for the recent period is yet to be released, which restricts further insights about the full resumption of services. Besides, it is worth reiterating that the key purpose of the implementation strategy was to generate demand by providing information to the pregnant women through various channels. Although in this study we do not evaluate the impact directly, previous studies indicate that counselling-based services are associated with improvement in maternal and child health outcomes (Nguyen *et al.*, 2019). The study thus provides encouraging insights into how the integration of this algorithmic approach can lead to an increase in skilled birth deliveries. This relationship also derives from a more fundamental fact that women who receive ANC services are more likely to delivery with a skilled birth attendant (Guliani *et al.*, 2012). Going forward, community health workers who are trained during this intervention can be deployed to provide counselling about dietary habits post-pregnancy and during post-natal care and family planning outreach. These steps will ensure that the women receive a continuum of service during pregnancy and in the postpartum period.

Integrated Child Development Services (ICDS) is the largest flagship programme of the Government of India for early childcare and development. In particular lessons learnt from this intervention could be useful for ensuring ICDS. However, departmental convergence and guidelines will have to be jointly issued to achieve this integration, as antenatal services are within the ambit of theNHM. Activity budgets in ICDS will need to be strengthened. The health sector budget is more evolved than ICDS budgets; also, the activities are more diverse in health. The information system and sharing has to improve between ICDS and the NHM; ideally, they should integrate to provide a clear continuum (International Food Policy Research Institute, 2018).

To conclude, the algorithm-based protocol seems an effective strategy that simplifies the workload of ANMs and ensures targeted delivery of services and counselling. Utilizing the pool of existing human resources at the facility level also qualifies this approach to be a rather cost-effect alternative. This integration brought renewed focus on the importance of routine ANC and the supply chain systems leading to improved service delivery and monitoring systems. The bigger contribution of this initiative has been its impact on policy discussions and among policy makers to explore options for provision of additional nutrition support to at-risk pregnant women, and also integrating anxiety/depression along with nutrition risks as part of high-risk pregnancy.

## Supplementary Material

czad011_Supp

## Data Availability

The study uses data from the factsheets of the fourth and fifth rounds of the National Family Health Survey (NFHS) which were conducted during 2015–16 and 2019–21respectively. The data are available from the DHS website on the following link National Family Health Survey (rchiips.org). The data for HMIS are available on HMIS-Health Management Information System (nhp.gov.in). Budget figures were obtained from UNICEF Madhya Pradesh based on Record of Proceedings, 2020–21, MOHFW. Data obtained from the Reproductive and Child Health Register will be made available on request.

## Reflexivity statement

The authors include seven females and six males and span multiple levels of seniority. All authors have engaged for several years in research activities with a health policy and systems focus, with four authors having done so for more than 10 years. Collectively, the authors have applied research experience in India. The authors’ academic training is diverse, with two being health economists, five with degrees in medicine, two with degrees in health management and four specializing in public health nutrition. All authors have several years’ experience conducting qualitative research and literature reviews.
