# Supplementary material for: Integrating an algorithmic and health systems thinking approach to improve the uptake of government antenatal nutrition services in Vidisha, Madhya Pradesh (India), 2018 to 2021
Source: Health Policy Plan. 2023 Feb 6;38(4):454–63. doi: 10.1093/heapol/czad011 (PMC10089058; doi:10.1093/heapol/czad011)
Supplement: czad011_Supp [file czad011_supp.zip › Supplementary file.docx]

**Table S1: External fund allocated by UNICEF for pilot implementation in Vidisha district, Madhya Pradesh, India FY 2019-20**

| SN | Budget Head | | Amount (USD) |
| --- | --- | --- | --- |
| 1 | Designing and Printing of Counselling material | | 10,162 |
| 2 | *Training cost* | | 13,225 |
| 2.1 | Sensitization workshop for district level functionaries of Vidisha district, select participants from Ujjain district (1st Nov 2018) for one day | | 1,328 |
| 2.2 | Two days training of Facility staff (7-8 and 12-13 Dec) for two batches | | 2,756 |
| 2.3 | One day orientation for block level ICDS supervisors (for estimated 95 participants) for two batches | | 1,211 |
| 2.4 | One day orientation for block level community volunteers (ASHA Sahyogi's) at district for 3 batches | | 2,250 |
| 2.5 | Two days training of outreach ANMs for six batches | | 5,680 |
| 3 | Monitoring and Handholding support | | 6,130 |
|  | **Total** |  | **29,518** |

Source: UNICEF Madhya Pradesh

(Exchange rate as per 9th Feb 2022 taken as 1 INR = 0.0134 USD, Source- India Foreign exchange rate

**Table S2: Human resource involved in the programme feasibility testing of the maternal nutrition algorithm Madhya Pradesh- pilot phase (2019) and expansion phase (2020-21)**

| District | District Hospital functionaries | | CH** | | CHC** | | Civil Dispensary / UPHC | PHC** | SHC** | Total |
| --- | --- | --- | --- | --- | --- | --- | --- | --- | --- | --- |
|  | Medical Officer* | Staff Nurse | Medical Officer | Staff Nurse | Nodal Maternal Health | SN | MO / SN | MO/ SN | ANM |  |
| *Pilot phase (2019)* | | | | | | | | | | |
| Vidisha | 1 | 2 | 2 | 4 | 7 | 11 | 0 | 25 | 174 | 226 |
| *Expansion phase (2020-21)* | | | | | | | | | | |
| Rajgarh | 1 | 2 | 3 | 3 | 6 | 12 | 2 | 27 | 168 | 224 |
| Barwani | 1 | 2 | 1 | 1 | 8 | 16 | 1 | 30 | 362 | 422 |
| Khandwa | 1 | 2 | 1 | 1 | 6 | 12 | 0 | 32 | 203 | 258 |
| Damoh | 1 | 2 | 1 | 1 | 6 | 12 | 0 | 15 | 192 | 230 |
| Chhatarpur | 1 | 2 | 0 | 0 | 10 | 20 | 1 | 37 | 257 | 328 |
| Singrauli | 1 | 2 | 0 | 0 | 6 | 12 | 0 | 15 | 227 | 263 |
| Guna | 1 | 2 | 1 | 1 | 5 | 10 | 1 | 15 | 176 | 212 |
| Pooled | 7 | 14 | 7 | 7 | 47 | 94 | 5 | 171 | 1582 | 1937 |
|  |  |  |  |  |  |  |  |  |  |  |
| Total (2019-21) | **8** | **16** | **9** | **11** | **54** | **105** | **5** | **196** | **1759** | **2163** |

* Medical Officer (MO) (Gynecologist, Lady Medical Officer).

**The primary healthcare system of India has three pillars Primary Health Centre (PHC), Community Health Centre(CHC) and Sub Centre(SC). SC is the first contact point between community and the primary health system. PHC aims to provide preventive and curative care. CHC serves as referral point for PHC and provide specialist care.

**Table S3: Socio-economic and maternal nutrition profile of Pilot district (Intervention Area 1), and Non-expansion district (Control)**

| Indicators | Vidisha | Ashok Nagar |  |
| --- | --- | --- | --- |
|  | (pilot intervention district) | (control district) |  |
| Total Population (Census, 2011) | 14,58,212 | 8,45,071 |  |
| % of ST Population (Census, 2011) | 4.63 | 9.71 |  |
| % of SC Population (Census, 2011) | 20.03 | 20.8 |  |
| Number of AWCs per 100,000 population | 132 | 108 |  |
| Number of SHC per 100,000 population | 14 | 13 |  |
| Number of Villages (Census, 2011) | 1614 | 899 |  |
| Childhood Stunting (NFHS-4, 2016) | 41.4 | 42.5 |  |
| Low BMI in women (NFHS-4, 2016) | 28 | 30.1 |  |
| High BMI in women (NFHS-4, 2016) | 11.3 | 10 |  |
| Anemia in women (NFHS-4, 2016) | 55.5 | 42.3 |  |
| Women with 10 or more years of schooling (NFHS-4, 2016) | 12.4 | 12.3 |  |
| Mothers who had at least 4 antenatal care visits (NFHS-4, 2016) | 16.9 | 38.5 |  |

**Table S4: Background characteristics of the respondents, Madhya Pradesh**

|  | NFHS-4 (2016) | | NFHS-5 (2021) | |
| --- | --- | --- | --- | --- |
|  | N | % | N | % |
| Sector |  |  |  |  |
| Rural | 3748 | 82.4 | 2559 | 81.4 |
| Urban | 802 | 17.6 | 583 | 18.6 |
| Age of women |  |  |  |  |
| 15-19 years | 119 | 2.6 | 69 | 2.2 |
| 20-24 years | 1728 | 38 | 1183 | 37.6 |
| 25-29 years | 1673 | 36.8 | 1292 | 41.1 |
| 30 years and above | 1030 | 22.6 | 598 | 19 |
| Education Respondent |  |  |  |  |
| No Schooling | 1986 | 43.6 | 864 | 27.5 |
| <5 years complete | 326 | 7.2 | 146 | 4.6 |
| 5-7 years complete | 917 | 20.2 | 579 | 18.4 |
| 8-9 years complete | 804 | 17.7 | 952 | 30.3 |
| 10-11 years complete | 209 | 4.6 | 180 | 5.7 |
| 12 or more years complete | 308 | 6.8 | 421 | 13.4 |
| Religion |  |  |  |  |
| Hindu | 4271 | 93.9 | 2955 | 94 |
| Muslim/others | 279 | 6.1 | 187 | 6 |
| Social group |  |  |  |  |
| SC | 827 | 18.2 | 613 | 19.5 |
| ST | 1090 | 24 | 679 | 21.6 |
| OBC | 2102 | 46.2 | 1348 | 42.9 |
| Other/DNK/Missing | 532 | 11.7 | 501 | 16 |
| Wealth |  |  |  |  |
| Lowest | 1939 | 42.6 | 1277 | 40.6 |
| Second | 1182 | 26 | 762 | 24.3 |
| Middle | 664 | 14.6 | 471 | 15 |
| Fourth | 441 | 9.7 | 372 | 11.8 |
| Highest | 323 | 7.1 | 260 | 8.3 |
| children |  |  |  |  |
| Up to 2 children | 2750 | 60.4 | 1974 | 62.8 |
| 3 to 4 children | 1391 | 30.6 | 973 | 31 |
| More than 4 children | 409 | 9 | 195 | 6.2 |
| District |  |  |  |  |
| Chhatarpur | 597 | 13.1 | 453 | 14.4 |
| Damoh | 437 | 9.6 | 355 | 11.3 |
| Barwani | 638 | 14 | 414 | 13.2 |
| Rajgarh | 554 | 12.2 | 387 | 12.3 |
| Vidisha | 584 | 12.8 | 396 | 12.6 |
| Guna | 524 | 11.5 | 366 | 11.6 |
| Ashoknagar | 280 | 6.1 | 222 | 7.1 |
| Singrauli | 492 | 10.8 | 301 | 9.6 |
| Khandwa (East Nimar) | 443 | 9.7 | 249 | 7.9 |
| Total | 4550 | 100 | 3142 | 100 |

**Table S5: Status of Maternal Malnutrition and Anemia in in Pilot district (Intervention Area 1), Expansion districts (Intervention Area 2) and Non-expansion district (Control) of M. Pradesh**

|  | **Women who are thin (BMI <18.5 kg/m2) (%)** | | **Women who are overweight or obese (BMI ≥25.0 kg/m2) (%)** | | **Women age 15-49 years who are anaemic (%)** | |
| --- | --- | --- | --- | --- | --- | --- |
|  | NFHS-4, 2016 | NFHS-5, 2021 | NFHS-4, 2016 | NFHS-5, 2021 | NFHS-4, 2016 | NFHS-5, 2021 |
| Vidisha (area 1) | 28 | 23.1 | 11.3 | 19.8 | 44.2 | 38.5 |
| Rajgarh (area 2) | 37.5 | 28 | 7.2 | 14.1 | 50.3 | 52.3 |
| Barwani (area 2) | 40.8 | 27.1 | 10.8 | 8.2 | 65.8 | 58.4 |
| Khandwa (area 2) | 34.7 | 21.7 | 12.9 | 13.7 | 58.5 | 64.8 |
| Damoh (area 2) | 27.1 | 23.7 | 13 | 17.4 | 45.5 | 48.1 |
| Chhatarpur (area 2) | 28.2 | 25.2 | 10.4 | 13.9 | 48.1 | 63.5 |
| Singrauli (area 2) | 19.4 | 25.6 | 11 | 11.9 | 52.6 | 54.1 |
| Guna (area 2) | 34.2 | 18.4 | 10.9 | 12 | 46.2 | 49.8 |
| Ashok Nagar district (control) | 30.1 | 26.1 | 10 | 15.4 | 42.3 | 46.1 |
| Difference -in-difference |  | -0.9 |  | 3.1 |  |  |

Note: Difference-in-difference calculated as net improvement in intervention area over control area between 2016-17 and 2021-22. Figures for did not provided for Ashok Nagar as anemia has increased

**Table S6: Status of Maternal Services in Pilot district (Intervention Area 1), Expansion districts (Intervention Area 2) and Non-expansion district (Control) of M. Pradesh, Rural area**

|  | **Mothers who had an antenatal check-up in the first trimester (%)** | | | | **Mothers who had at least 4 antenatal care visits (%)** | | | | **Consumption of iron folic acid for 180 days or more in pregnancy (%)** | | | |
| --- | --- | --- | --- | --- | --- | --- | --- | --- | --- | --- | --- | --- |
|  | NFHS-4, 2016 | | NFHS-5, 2021 | | NFHS-4, 2016 | | NFHS-5, 2021 | | NFHS-4, 2016 | | NFHS-5, 2021 | |
|  | % | 95% CI | % | 95% CI | % | 95% CI | % | 95% CI | % | 95% CI | % | 95% CI |
| Chhatarpur | 34.9 | [29.0,41.3] | 66.2 | [58.3,73.2] | 15 | [10.6,20.7] | 35.7 | [28.9,43.1] | 3.3 | [1.7,6.4] | 12.5 | [7.3,20.5] |
| Damoh | 25.1 | [18.2,33.7] | 64 | [54.9,72.1] | 21.3 | [15.8,28.1] | 47.2 | [37.6,57.0] | 7.1 | [4.2,11.9] | 30.5 | [24.1,37.8] |
| Barwani | 36.6 | [28.2,45.8] | 89.3 | [83.9,93.0] | 22.8 | [15.9,31.5] | 63.3 | [55.4,70.5] | 11.5 | [7.4,17.2] | 31.2 | [22.2,41.9] |
| Rajgarh | 53.4 | [45.4,61.2] | 72.1 | [63.9,79.0] | 33.7 | [25.2,43.4] | 48.7 | [39.9,57.6] | 4.8 | [2.9,7.8] | 28.8 | [22.0,36.8] |
| Vidisha | 26.8 | [19.8,35.2] | 84.3 | [78.8,88.5] | 16.9 | [11.6,24.0] | 52.4 | [40.1,64.5] | 3.4 | [1.6,6.8] | 23.1 | [15.7,32.6] |
| Guna | 58 | [48.4,67.1] | 86.9 | [79.3,92.0] | 31.4 | [22.9,41.4] | 68.6 | [61.2,75.2] | 3.1 | [1.0,9.1] | 41.2 | [32.8,50.0] |
| Ashoknagar | 63.5 | [53.7,72.4] | 78.8 | [69.4,85.9] | 39.2 | [28.4,51.2] | 59.5 | [48.1,70.1] | 5.9 | [3.1,11.1] | 28.7 | [21.0,38.0] |
| Singrauli | 22.5 | [16.6,29.6] | 72.8 | [61.5,81.7] | 15 | [10.2,21.6] | 58.2 | [47.2,68.5] | 1.7 | [0.6,5.0] | 22.8 | [15.0,33.2] |
| Khandwa (East Nimar) | 70.6 | [63.4,76.8] | 65.3 | [51.5,76.9] | 45 | [37.2,53.1] | 59.9 | [44.8,73.4] | 8.9 | [5.7,13.8] | 26.9 | [16.8,40.1] |
| Unadjusted Difference -in-difference |  |  | 42.01*** |  |  |  | 13.85** |  |  |  | -3.72 |  |
| Adjusted Difference -in-difference |  |  | 40.69*** |  |  |  | 11.30* |  |  |  | -4.83 |  |

Note: Difference-in-difference calculated as net improvement in intervention area over control area between 2016-17 and 2021-22. Figures in parenthesis are 95% confidence interval. Level of significance-*p < .05. **p < .01. ***p < .001.

**Table S7: Status of Maternal Services in Pilot district (Intervention Area 1), Expansion districts (Intervention Area 2) and Non-expansion district (Control) of M. Pradesh, Urban area**

|  | **Mothers who had an antenatal check-up in the first trimester (%)** | | | | **Mothers who had at least 4 antenatal care visits (%)** | | | | **Consumption of iron folic acid for 180 days or more in pregnancy (%)** | | | |
| --- | --- | --- | --- | --- | --- | --- | --- | --- | --- | --- | --- | --- |
|  | NFHS-4, 2016 | | NFHS-5, 2021 | | NFHS-4, 2016 | | NFHS-5, 2021 | | NFHS-4, 2016 | | NFHS-5, 2021 | |
|  | % | 95% CI | % | 95% CI | % | 95% CI | % | 95% CI | % | 95% CI | % | 95% CI |
| Chhatarpur | 41.7 | [31.7,52.4] | 64.7 | [50.5,76.6] | 37.8 | [20.8,58.6] | 40.3 | [25.9,56.7] | 5.9 | [2.7,12.6] | 24.7 | [10.3,48.4] |
| Damoh | 52.8 | [35.2,69.7] | 77.6 | [57.9,89.8] | 35 | [24.4,47.2] | 43.9 | [29.7,59.2] | 8.2 | [2.1,27.1] | 24.2 | [11.0,45.3] |
| Barwani | 87.4 | [63.1,96.5] | 86.6 | [61.2,96.4] | 51.9 | [33.5,69.8] | 71 | [51.9,84.8] | 19.7 | [8.2,40.4] | 42.1 | [32.7,52.1] |
| Rajgarh | 45.6 | [29.7,62.3] | 88.1 | [64.0,96.9] | 48.3 | [16.5,81.5] | 83.3 | [49.7,96.2] | 8.9 | [2.3,28.5] | 27.1 | [14.0,46.1] |
| Vidisha | 38.5 | [20.8,59.8] | 87.3 | [64.1,96.4] | 16.9 | [5.0,44.3] | 62.7 | [38.1,82.1] | 5.9 | [1.3,22.8] | 32.3 | [18.4,50.2] |
| Guna | 68.4 | [50.3,82.3] | 77.2 | [58.3,89.2] | 33.4 | [20.6,49.4] | 68.8 | [54.6,80.1] | 6 | [2.4,14.4] | 50.9 | [35.6,66.0] |
| Ashoknagar | 88.2 | [75.9,94.6] | 94.1 | [80.2,98.4] | 35.6 | [18.2,57.9] | 53.4 | [28.1,77.0] | 12.2 | [4.5,29.2] | 17.5 | [8.2,33.3] |
| Singrauli | 63.3 | [40.5,81.4] | 73.9 | [57.6,85.5] | 51.2 | [35.7,66.4] | 57.3 | [34.3,77.5] | 1.9 | [0.3,12.2] | 15.6 | [6.5,32.9] |
| Khandwa (East Nimar) | 82.8 | [72.5,89.7] | 66.4 | [29.0,90.6] | 63 | [46.7,76.7] | 71.2 | [34.5,92.1] | 10.4 | [4.5,22.5] | 30.2 | [3.9,82.1] |
| Unadjusted Difference -in-difference |  |  | 35.89*** |  |  |  | 23.35* |  |  |  | 20.46** |  |
| Adjusted Difference -in-difference |  |  | 34.00*** |  |  |  | 15.97 |  |  |  | 19.79* |  |

Note: Difference-in-difference calculated as net improvement in intervention area over control area between 2016-17 and 2021-22. Figures in parenthesis are 95% confidence interval. Level of significance-*p < .05. **p < .01. ***p < .001.

**Table S8: Percentage change in indicators obtained from RCH register Program data**

| District Name | IFA 180 Tablets | Folic Acid 30 Tablets | Albendazole 1 Tablet | Calcium 180 Tablets |
| --- | --- | --- | --- | --- |
| AARR |  |  |  |  |
| East Nimar (Khandwa) | 32.6 | 21.6 | 172.7 | 77.6 |
| Chhatarpur | 38.2 | 40.0 | 178.6 | 302.9 |
| Guna | 200.0 | 214.8 | 223.3 | 271.7 |
| Rajgarh | 113.6 | 121.3 | 129.0 | 75.4 |
| Barwani | 54.6 | 67.3 | 120.9 | 280.4 |
| Singrauli | 36.1 | 16.0 | 200.3 | 241.5 |
| Ashoknagar | 34.4 | 15.0 | 184.0 | 163.9 |
| Damoh | 123.1 | 178.8 | 157.7 | 199.4 |
| Vidisha | 32.6 | 163.6 | 109.3 | 81.8 |

**Figure S1 Maternal Nutrition Algorithm for strengthening maternal nutrition services for pregnant women attending PMSMA/ANC**

Source: <http://nceard.roshni-cwcsa.co.in/UploadPDF/Algorithm_VHSND.pdf> .

**Figure S2 Maternal Nutrition Algorithm for strengthening Maternal nutrition services for pregnant women attending ANC**

Source: <http://nceard.roshni-cwcsa.co.in/UploadPDF/Algorithm_ANC%20OPD.pdf>
